# Autogenous *Streptococcus suis* serotype 1 bacterin: immunogenicities of sow and gilt vaccination protocols

**DOI:** 10.1186/s40813-025-00453-6

**Published:** 2025-07-18

**Authors:** Georg Freymüller, Silke Lehnert, Christine Unterweger, Thomas Voglmayr, Christoph G. Baums, Leonie Mayer

**Affiliations:** 1 Institute of Bacteriology and Mycology, Centre for Infectious Diseases, Faculty of Veterinary Medicine, Leipzig University, An den Tierkliniken 29, 04103 Leipzig, Germany; 2 Clinical Department for Farm Animals and Food System Science, Clinical Centre for Population Medicine in Fish, Pig and Poultry, University of Veterinary Medicine, Vienna A‑1210, Vienna, Austria; 3 Traunkreis Vet Clinic, Ried im Traunkreis, A-4551 Austria; 4 Aulendorf State Veterinary Diagnostic Centre, 88326 Aulendorf, Germany

**Keywords:** IgA, IgG, Maternally-derived antibodies, Colostrum, Intranasal vaccination

## Abstract

**Background:**

Diseases caused by *Streptococcus suis* (*S. suis*) infection have a major impact on return on investment, use of antibiotics and animal welfare in piglet rearing worldwide. *S. suis* bacterins are commonly used as autogenous vaccines in different countries but experimental studies indicate limitations of bacterins in protective efficacy. In this study we read out levels of IgG, IgM and IgA binding to a *S. suis* serotype (*cps*) 1 outbreak strain and bactericidal immunity after application of a homologous bacterin to sows and gilts. In the first trial we recorded immunogenicities after preparturient sow vaccination. In the second trial we compared an intranasal versus an intramuscular second boost application in gilts, and in the third trial the impact of the second boost application prior farrowing of gilts was specifically investigated.

**Results:**

Preparturient intramuscular application of a *S. suis cps*1 (*sly*^+^, *mrp*^+^, *epf*^+^) autogenous bacterin elicited significantly increased levels of serum IgG but not IgM binding to the surface of the homologous strain. Accordingly, specific serum IgG levels were significantly increased in the second and fourth week of life in piglets of these sows. Increased IgG levels were associated with decreased proliferation of *S. suis cps*1 in blood of 2-week-old piglets reared by vaccinated sows. The increase of IgM binding to *cps*1 between the fourth and sixth week of life was comparable between piglets farrowed by vaccinated and non-vaccinated sows. Levels of serum IgA binding to *S. suis cps*1 were not different between piglets fostered by vaccinated and non-vaccinated sows. Between the fourth and sixth week of life we recorded a significant increase in specific serum IgA levels. Intramuscular prime boost vaccination of gilts during quarantine elicited significantly increased specific serum IgG but not IgA levels. Levels of IgG in colostrum binding to *S. suis cps*1 were significantly increased only in gilts boostered intramuscularly 3 weeks pre farrowing and not in gilts boostered intranasally. Neither intramuscular nor intranasal boostering was associated with increased levels of specific IgA in colostrum. The significant influence of the second intramuscular boost vaccination pre farrowing in gilts on IgG levels in colostrum and in blood of 2-week-old-piglets was confirmed in the last trial.

**Conclusions:**

Intramuscular prime-boost vaccination of sows and gilts with an autogenous *S. suis cps*1 bacterin is associated with significantly increased levels of specific IgG in their colostrum and serum of 2- and 4-week-old piglets based on the investigations in one herd that had experienced a severe *S. suis cps*1 outbreak. After prime-boost vaccination during quarantine gilts should be boostered again pre-farrowing to ensure increased IgG levels in their piglets. A way to elicit increased specific IgA levels in colostrum, milk or serum through intramuscular or intranasal bacterin application was not identified.

## Background

*Streptococcus suis* (*S. suis*) causes arthritis, meningitis, septicemia and endocarditis in piglets of different ages. Heterogenicity in composition of the capsule as well as surface-associated and secreted factors, such as muramidase-released protein (MRP, gene *mrp*) and extracellular factor (EF, gene *epf*), respectively, is a hallmark of this major porcine pathogen [1, 2]. This is associated with substantial differences in virulence ranging from strains that mainly colonize mucosal surfaces to highly virulent strains causing outbreaks with high mortality [3]. Strains of clonal complex (CC) 1 are very virulent as they are involved in animal health problems worldwide and reproducibly cause disease in experimental infection [4, 5]. They mainly belong to *cps*2 and to a lesser extent also to *cps*1 and other serotypes.

Autogenous bacterins are commonly used in the field as no commercially available vaccine protects against different serotypes [6]. Different laboratories have confirmed that prime-boost vaccination with *cps*2 bacterins might elicit protection against the homologous strain [7–9]. However, priming with a *cps*2 bacterin alone does not result in protection [10]. Protective efficacy depends on the adjuvant and the vaccination protocol [7, 9]. Prime-boost vaccination of preparturient sows with a *cps*2 autogenous bacterin elicited protection against intravenous homologous challenge in 6-week-old but not in 8-week-old littermates [11]. Bacterins of *cps*9 are considered to be less protective [12, 13].

Intranasal application of a *S. suis* vaccine has only been investigated in very few studies [14]. In mice, intranasal immunization with a recombinant vaccine consisting of five surface-associated or secreted factors elicited increased specific serum IgG levels, increased systemic and mucosal specific IgA levels and antigen-specific IL-17 A-secreting splenocytes. This was associated with protection against systemic disease and also reduction of nasopharyngeal colonization [15]. Brockmeier et al. [16] vaccinated piglets simultaneously intranasally and intramuscularly with a different recombinant multicomponent vaccine. Protection against intranasal challenge with a *cps*2 strain of CC 1 was recorded. However, increased levels of antigen-specific IgA have not been described in any *S. suis* vaccination study in pigs.

Here we report the results of three field studies designed to read out maternally-derived IgG and IgA antibodies after different vaccination schedules using an autogenous *cps*1 bacterin. Intranasal boost immunization pre-farrowing was evaluated as an alternative application route in one trial. Vaccination of gilts during quarantine in combination with booster immunization pre-farrowing was specifically investigated in this study, as this is highly relevant to the field.

## Materials and methods

### History and status of the herd

An Austrian breeding farm of 160 sows with a share of 50% Austrian large white and 50% Austrian F1 (Austrian large white sow x Austrian Landrace boar) sows was managed over many years in the past as a closed herd and with a 3-week-production-rhythm. Based on regularly breeding herd investigations and sampling of 20% of sold gilts, the PRRSV status was considered unsuspicious. The herd was free of clinical signs of atrophic rhinitis and mange. In August 2017 ten gilts were introduced into the herd within a genetic exchange project. Prior to the introduction, these gilts were clinically unsuspicious over a 4 weeks quarantine period when they were vaccinated against parvovirosis, PCV-2 associated diseases, influenza A, erysipelas and enzootic pneumonia. Two months prior to the *S. suis* outbreak described under Results numerous 2-week-old suckling piglets showed dyspnoe and arthritis (January 2018).

### Necropsies and pathohistological investigations

Necropsies and pathohistological investigations of diseased piglets were conducted by the Institute of Pathology of the University of Veterinary Medicine Vienna as regular diagnostic service.

### Design of the vaccination experiments

**Fig. 1:**
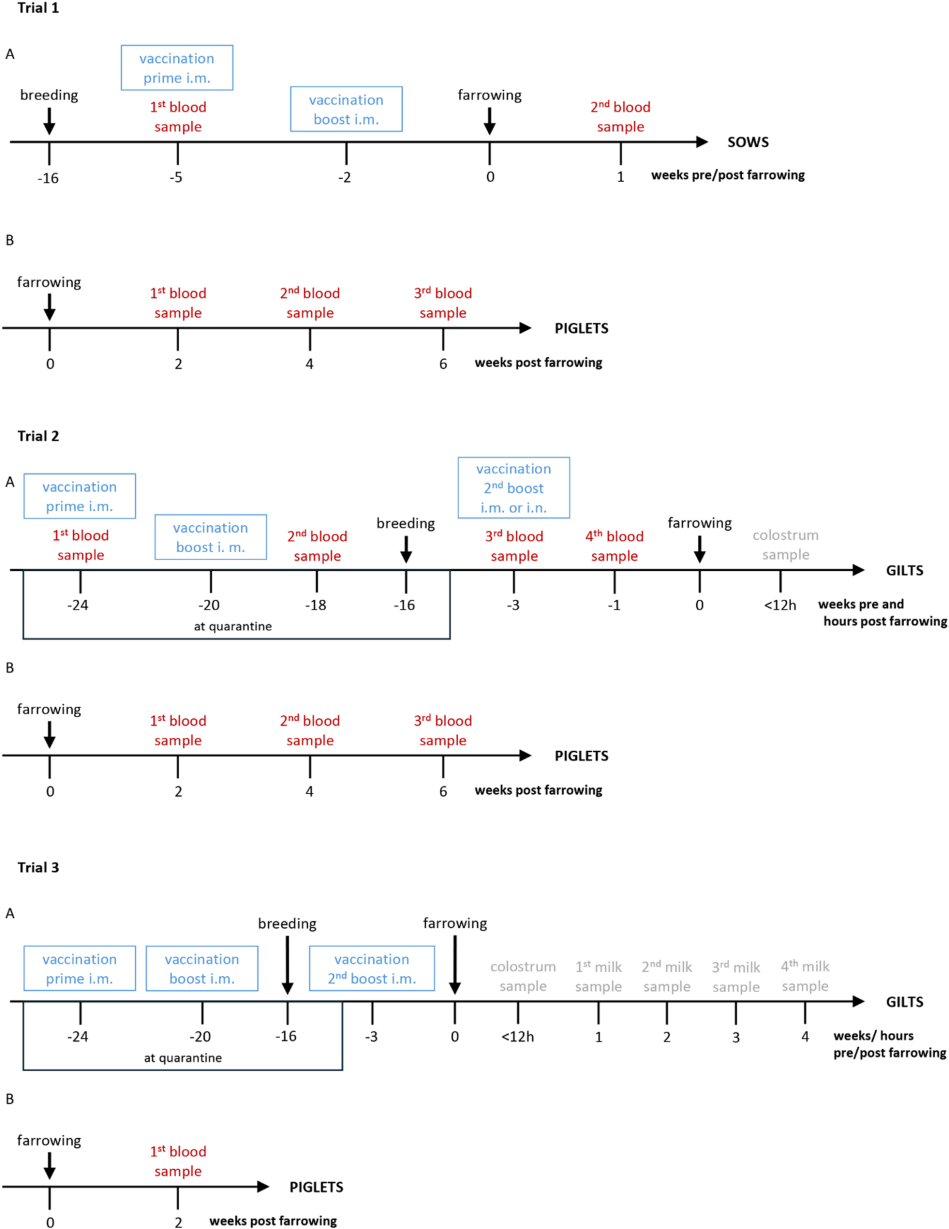
Experimental design of the 3 trials with **A** depicting time line of vaccination and sampling of sows (trial 1) or gilts (trials 2 and 3) and **B** indicating time points of blood sampling in their piglets (3 (trial 1) or 2 (trials 2 and 3) per litter treated as triplicate or duplicate, respectively). In trials 1 and 3 vaccination was conducted through intramuscular application of an autogenous *S. suis cps*1 bacterin including Emulsigen^®^ as adjuvant. In trial 2, the 1st and 2nd vaccination (prime-boost) was also done the same way but in the 3rd vaccination (2nd boost) the autogenous *S. suis cps*1 bacterin including Montanide™ Gel 02 PR as adjuvant was applied either intramuscularly or intranasally (both adjuvants with a final concentration of 10%).

Suckling piglets born by different sows or gilts were not cross-fostered on this farm in any of the three trials. The time points of vaccination and sampling of sows, gilts and piglets in the three trials is depicted in Fig. 1.

In the first trial 5 sows were prime-boost-vaccinated intramuscularly five and two weeks *pre partum* with an autogenous *cps*1 bacterin including 10% Emulsigen^®^ (oil-in-water emulsion, MVP Adjuvants, Phibro Animal Health Corporation, Teaneck, NJ, USA) as adjuvant. No *S. suis* vaccine was applied to five control sows of the same group. Serum samples of these 10 sows were taken pre immunization and one week after farrowing (3 weeks after boost vaccination). The sows of the first trial were already present in the herd during the described disease outbreak. From each investigated litter, three healthy and well-developed piglets were selected randomly for this study and received individual ear-tags. Serum samples were collected at the age of 2, 4 and 6 weeks of life. Due to the death of 2 piglets, only 13 piglets of vaccinated sows were investigated at an age of 6 weeks in the first trial.

In the second trial, three groups of gilts were set up (a total of 27 gilts). Two groups were prime-boost vaccinated intramuscularly with an autogenous *cps*1 bacterin (adjuvant 10% Emulsigen^®^) during quarantine. The interval between prime and the first boost was 4 weeks. Both vaccinated groups obtained a reproduction-related boost immunization 3 weeks prior to farrowing with the same inactivated *cps* 1 strain but a different adjuvant (10% Montanide™ Gel 02 PR, sodium polyacrylate-in-water, SEPPIC, Fairfield, NJ, USA). One group was vaccinated intramuscularly (i.m. group, *n* = 8) and the other group only intranasally (i.m. + i.n. group, *n* = 9). The third group of gilts obtained no vaccination (control, *n* = 10). Serum samples of gilts were drawn prior priming and 2 weeks after each boost immunization (the last one approximately one-week prior farrowing). Colostrum samples of 6 mL were collected within 12 h after the onset of birth after dry cleaning of the udder. Serum samples of two piglets from each litter were collected at the age of 2, 4 and 6 weeks of life. At the age of 2 weeks, heparinized blood was also taken from the piglets and a bactericidal assay was conducted.

In the third trial, gilts (*n* = 13) were prime-boost vaccinated intramuscularly with the autogenous *cps*1 vaccine including 10% Emulsigen^®^ at intervals of four weeks during quarantine (same vaccine as in trial 1). Three weeks *pre partum* one group obtained an intramuscular second boost (*n* = 7) with the same vaccine while the other group (*n* = 6) remained non-boostered. Colostrum samples were collected again within 12 h after birth, milk samples were collected 1, 2, 3 and 4 weeks *post partum*. Additionally, blood samples from piglets (*n* = 2/gilt) were drawn at an age of 2 weeks of life.

### Vaccine

The *S. suis cps*1 bacterin used as autogenous vaccine was provided by BS-Immun GmbH (Mödling, Austria). In all trials the vaccine formulation of the prime-boost immunization consisted of formalin-inactivated streptococci only (10^9^ colony-forming units (CFU) *S. suis cps*1 per dose) mixed with 10% Emulsigen^®^. In trial 2 the vaccine formulation of the second booster vaccination consisted of formalin-inactivated streptococci (10^9^ CFU *S. suis cps*1 per dose) mixed with Montanide™ Gel 02 PR. All procedures for vaccine formulations with tested adjuvants were carried out according to the manufacturer’s protocols.

### Immunization and sampling

The autogenous *S. suis cps*1 vaccine with Emulsigen^®^ as adjuvans was administered in a volume of 2 mL per dose intramuscularly into the neck muscles. The autogenous *S. suis cps*1 + Montanide™ Gel 02 PR vaccine was either administered in a volume of 2 mL per dose intramuscularly or intranasally (1 mL per nostril). Intranasal application was conducted after restraining the gilts with a wire rope using the applicator Bovalto Breezer (Boehringer Ingelheim, internal number 51202691). The nostril was completely covered with a rubber lip and the vaccine was sprayed with a droplet size of 30 μm to 100 μm.

The sampling times of sows, gilts and piglets are shown in Fig. 1. The blood sample (10 mL) was obtained from the jugular vein using a 14 g x 2.5-inch needle for sows and gilts and a 18 g x 1.5-inch needle for piglets. To take samples, the gilts were restrained using an upper jaw sling and the piglets were fixed in the supine position. Serum was collected following centrifugation and stored at -18 °C until analysis.

After dry cleaning of the udder colostrum samples of 6 mL were collected within 12 h after the onset of birth. Colostrum was collected once from all teats and combined. After injection of 15 I.E. oxytocin and dry cleaning of the udder, milk samples were taken from all teats and combined.

### Genotyping of *S.°suis*

Profiling of virulence-associated genes, sequencing of the *cps*K gene and determination of the sequence type of *S. suis* isolates of this herd (namely 2093/1; 2095/1; 2402/1; 2303/1 and 2400/1) was conducted already in a previous study (see Supplementary Material in [17]).

### Bacterial strains and growth conditions

Streptococci were cultured on Columbia agar plates with 6% sheep blood or in BactoTM Todd Hewitt Broth (THB) under microaerophilic conditions (5% CO_2_). Strains were stored at -80 °C in 1 mL THB with 20% glycerine.

### Bactericidal assay

In bactericidal assays, heparinized blood is infected with *S. suis in vitro* to read out control of bacterial survival by opsonophagocytosis. In the 2^nd^ trial, heparinized blood was collected from 2 piglets per litter at 2 weeks of age. Bactericidal assays were performed and conducted as described before [18]. Briefly, 500 µL freshly drawn porcine heparinized (16 I. U. heparin/ml) blood was inoculated with 3 × 10^6^ CFU of *S. suis* strain 2093/1. Samples were incubated on a rotator for 120 min at 37^°^C. To determine CFU serial dilutions of samples collected at t = 0 min and t = 120 min were plated on blood agar plates. Survival factors were calculated dividing CFU at t = 120 min by CFU at t = 0 min.

### Bactericidal assay with reconstituted blood

In this assay blood was reconstituted with serum drawn from piglets nursed by vaccinated versus non-vaccinated sows. Specifically, samples consisted of reconstituted blood containing 100 µL serum of the animal to be investigated and 100 µL blood cells of pigs from a different herd. Reconstituted blood samples were inoculated with 2.4 × 10^4^ CFU of *S. suis* strain 2093/1 and incubated rotating at 37 °C for 2 h. CFUs were determined through serial dilutions of samples collected at t = 0 min and t = 120 min. Survival factors were calculated dividing CFU at t = 120 min by CFU at t = 0 min.

### Detection of α-*cps*1-IgG, α-*cps*1-IgM and α-*cps*1-IgA in serum and milk samples

IgG, IgM and IgA levels binding to the outbreak strain were essentially determined as described previously [17] with the following modifications. For detection of IgG, IgA and IgM antibodies Nunc MaxiSorp™ flat-bottom plates (Fisher Scientific, Nunc A/S, Roskilde, Denmark) were coated with 0.2% formaldehyde-inactivated bacteria of strain 2093/1 (*cps*1) overnight at 4^◦^C. Plates were blocked with 0.5% bovine serum albumin (BSA) and 0.1% gelatin in PBS. After washing, serum samples were added in duplicates serially diluted in PBS with 0.5% bovine serum albumin (BSA), 0.1% gelatin and 0.05% Tween20.

For detection of IgG and IgM, a secondary polyclonal peroxidase-conjugated goat anti-pig IgG (A100-105 P, Bethyl, Montgomery, Texas, 1:1000) and goat-anti-porcine-IgM horseradish-peroxidase (HRP) conjugated (NBP2- 42699 H, Novus Biologicals, Centennial, USA, 1:10.000) antibody was used, respectively. A pool of 5 sera of convalescent piglets which had survived an experimental *cps*14 challenge was used as a standard defined to contain 100 ELISA units. Plates were developed using 2.2-azino-di-(3-ethylbenzithiazoline sulfonate) (ABTS, Roche, Merck, Darmstadt, Germany) and H_2_O_2_ as the substrate. Absorbance was measured at 405 nm.

For detection of IgA, a peroxidase-conjugated goat anti-pig IgA (A100-102 P, Bethyl, Montgomery, Texas, 1:5000) was used. The mentioned pool of 5 convalescent sera served also as standard defining 100 ELISA units in the IgA ELISA. Plates were developed using 3,3′,5,5′-tetramethylbenzidin (Merck, Darmstadt, Germany) and H_2_O_2_ as the substrate. The reaction was stopped with 1 M sulphuric acid. Absorbance was measured at 450 nm.

### Statistical analysis

Normal distribution was evaluated using the Shapiro-Wilk-test. According to this, differences between two groups were analyzed with the Mann-Whitney *U*-test or the unpaired t-test and comparison of time point values within the same group in case of no more than two repeated measures were carried out using the Wilcoxon test or the paired t-test. The evaluation of more than two time points was carried out using two-way analysis of variance (ANOVA) or Kruskall-Wallis with a subsequent Tukey’s or Dunn’s multiple comparisons test, respectively. From each litter three (trial 1) or two (trial 2 and trial 3) piglets were sampled. For determination of specific antibody levels, the sera drawn from littermates at the same time point were considered a triplicate or duplicate, respectively, because sow or gilt vaccination was investigated. For presentation of data on bacterial survival individual values were used. Means and standard deviations or medians and quartils are shown as indicated in the different figures.

All statistical tests were conducted with GraphPad Prism 10.2.3 software. Probabilities lower than 0.05 were considered significant (*p < 0.05, **p < 0.01, ***p < 0.001, ****p < 0.0001).

## Results

### Description of a *S. suis cps* 1 outbreak in suckling piglets

In March 2018, the herd experienced an outbreak of devastating disease in 2- to 3-week-old suckling piglets. In the majority of litters (*n* = 25) two to three piglets showed signs of central nervous system dysfunction. None of the litters remained unaffected. In three litters, all piglets were unable to stand up. Fever, swollen joints, opisthotonus convulsions and nystagmus were common clinical signs. During this outbreak, five severely diseased piglets with clinical signs of central nervous system dysfunction and swollen joints were sacrificed and screened for causative pathogens. In two piglets, a high-grade of predominantly purulent meningitis with localised fibrin effusions in the meninx was confirmed pathohistologically. *S. suis* was detected in all five animals in brain and/or joint swabs. Two invasive isolates from each of the five piglets were differentiated further by PCR and sequencing. All ten isolates had a *sly*+, *mrp*+, *epf* + *cps*1 genotype and belonged to sequence type 1 [17]. Strain 2093/1, originating from the meninx of a diseased piglet [17], was used as a representative of these isolates in the following laboratory tests. During the outbreak, sick piglets were initially treated with amoxicillin (15 mg per kg bodyweight) and dexamethasone (0.06 mg per kg bodyweight) intramuscularly. Subsequently, piglets were treated intramuscularly with ceftiofur (5 mg per kg bodyweight) 24 h after birth and on the 6th day of life (as metaphylaxis). Vaccination with an autogenous bacterin containing the outbreak strain and 10% Emulsigen^®^ as adjuvant was implemented in mother sows.

### Trial 1: Preparturient prime-boost vaccination of sows

**Fig. 2:**
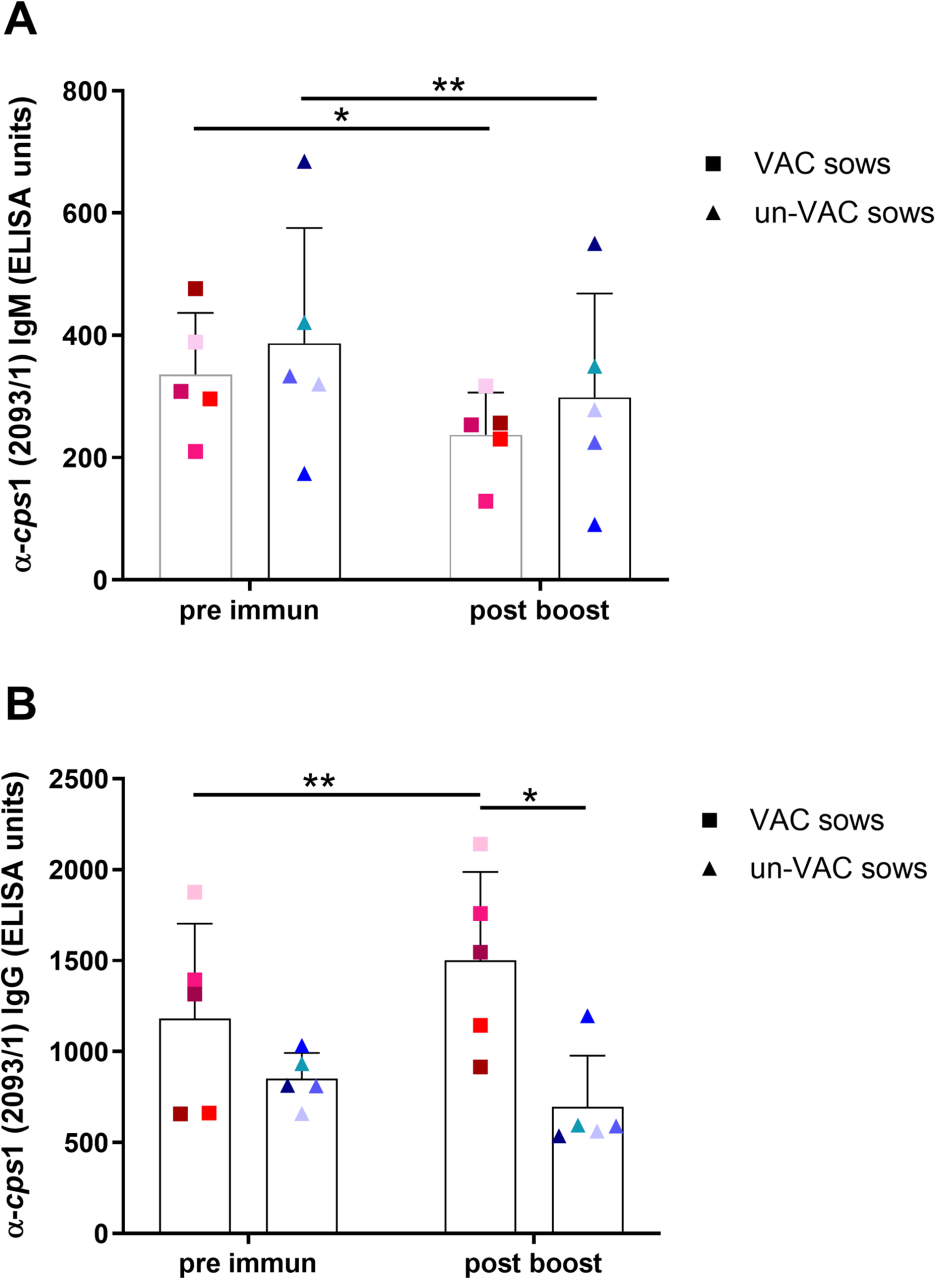
Prime-boost immunization of sows with autogenous *cps*1 bacterin induces significant higher levels of IgG **(B)** but not IgM **(A)** antibodies three weeks post boost in comparison to unvaccinated sows (trial 1). In trial one, sows (*n* = 5) were prime-boost-vaccinated 5 and 2 weeks before farrowing with an autogenous *cps*1 sequence type 1 (strain 2093/1) bacterin (VAC sows) or no vaccination was administered (un-VAC sows, *n* = 5). A specific sow is indicated by the same color in all diagrams. Mean values are indicated by horizontal lines, standard deviations by error bars. Statistical analyses were conducted with the Mann-Whitney *U*-test (comparison between groups at time point post boost), the Wilcoxon test (comparison of time points within group control sows) and the paired t-test (comparison of time points within group immunized sows). Significant differences are indicated (* *p* < 0.05, ** *p* < 0.01)

In the first trial, serum IgG and IgM binding to the *S. suis cps*1 outbreak strain were determined in 5 vaccinated and 5 non-vaccinated sows. Vaccination was conducted 5 and 2 weeks *pre partum* with an autogenous *cps*1 bacterin. Prime-boost immunization led to a significant increase of IgG-α-*cps*1 (2093/1) antibodies in immunized sows with means of 1181 ELISA units (S.D. = 522 ELISA units) pre immunization and 1502 ELISA units (S.D. = 487 ELISA units) post boost whereas no significant change in IgG levels of control sows was observed (Fig. 2B). Around time of farrowing (post boost) IgG levels were significantly higher in immunized sows compared to the control group (mean = 697 ELISA units, S.D. = 281 ELISA units). No significant differences between the groups were recorded for IgM (Fig. 2A).

**Fig. 3:**
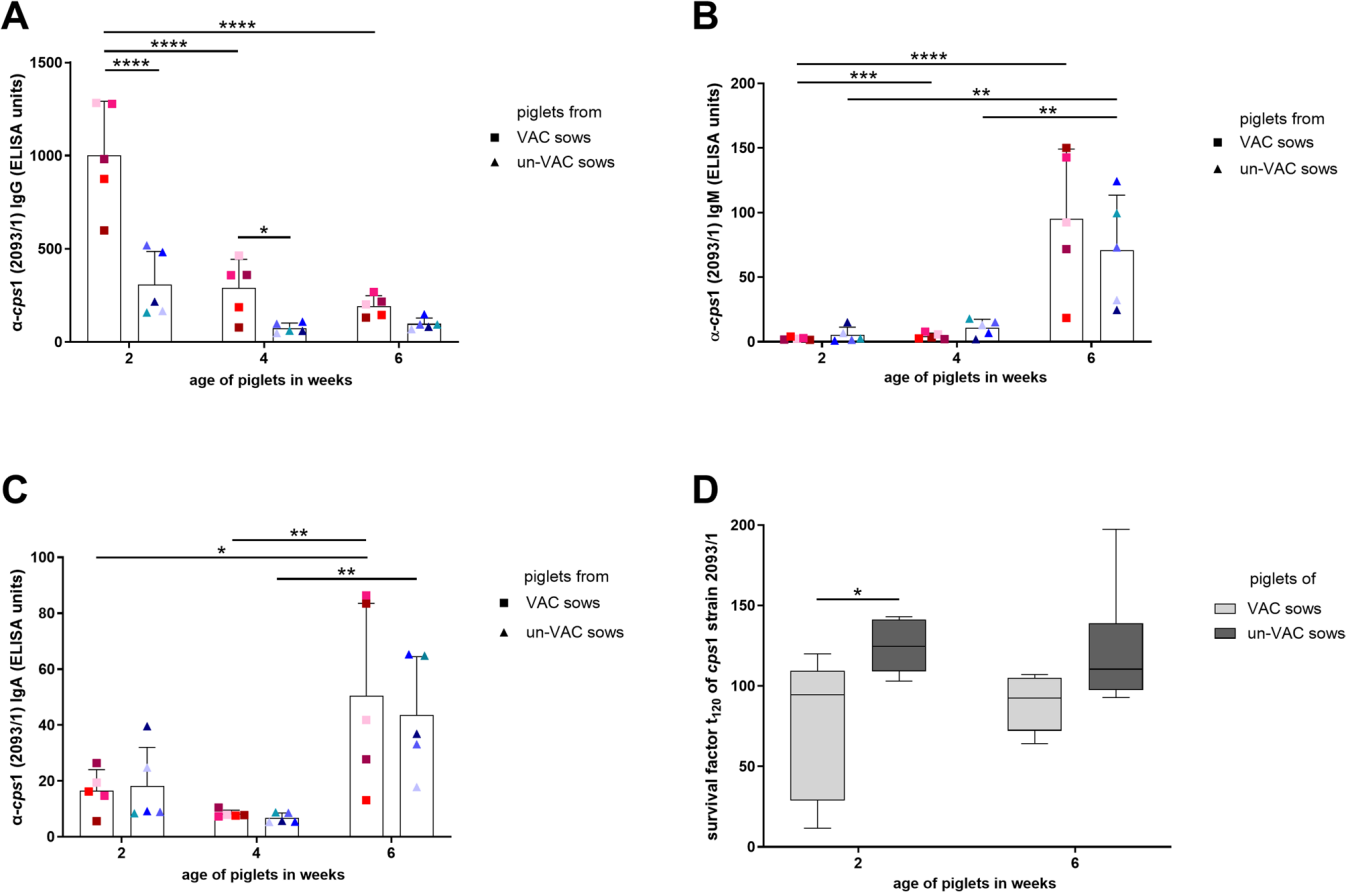
Levels of IgG **(A)**, IgM **(B)** and IgA **(C)** antibodies binding to *S. suis cps*1 in serum samples drawn from piglets fostered by immunized (VAC sows) or non-immunized sows (un-VAC sows) and survival factors of *S. suis cps*1 in bactericidal assay with blood reconstituted with the respective serum samples as indicated **(D)** (trial 1). Piglets were raised by sows either vaccinated with an autogenous *S. suis cps*1 bacterin 5 and 2 weeks pre farrowing or not vaccinated at all (*n* = 5 sows/group). Three individuals from each litter were chosen randomly for the experiments. Serum samples were collected from the same piglets at the age of 2, 4 and 6 weeks of life (*n* = 15/group, *n* = 3/litter). **A**,** B**,** C** Levels of IgG (Fig. 3A), IgM (Fig. 3B) as well as IgA (Fig. 3C) against *cps*1 strain 2093/1 in serum samples were determined in a whole cell ELISA. Litter means were calculated and used as statistical parameter. A specific litter is indicated by the same color in all diagrams. Mean values are indicated by horizontal lines, standard deviations by error bars. Statistical analyses were conducted with the two-way ANOVA and a subsequent Tukey’s multiple comparisons test. Significant differences are indicated (* *p* < 0.05, ** *p* < 0.01, *** *p* < 0.001, **** *p* < 0.0001). **D** To determine survival of *S. suis cps*1 in blood in the presence of antibodies of piglets, blood was reconstituted with serum drawn at the indicated time points of life and porcine blood cells. Subsequently, the reconstituted blood was inoculated with 2.4 × 10^4^ CFU of the outbreak strain (Fig. 3D). Samples were incubated for 2 h and bacterial contents were determined. Survival factors were calculated dividing CFU at t = 120 min by CFU at t = 0 min. Medians and quartiles are indicated by horizontal lines. Statistical analyses were conducted with the Mann-Whitney *U*-test (comparison between groups) and the Wilcoxon test (comparison of time points within group). Significant difference is indicated (* *p* < 0.05)

A significant decrease of specific IgG levels was recorded in piglets of vaccinated and non-vaccinated sows between 2 and 6 weeks of age (Fig. 3A). Piglets reared by vaccinated sows showed significantly higher levels of specific IgG at an age of 2 and 4 weeks (1003.4 ELISA units, S.D. = 288.9 and 289.8 ELISA units, S.D. = 154.1 ELISA units, respectively) compared to individuals in the control group (308.8 ELISA units, S.D. = 177.3 and 75.6 ELISA units, S.D. = 26.6 ELISA units, respectively). The specific IgG levels of piglets from vaccinated sows were also higher at the age of 6 weeks with a litter mean of 193.0 ELISA units (S.D. = 55.8 ELISA units) in comparison to the control piglets with 98.2 ELISA units (S.D. = 30.4 ELISA units), but differences were not significant anymore, most likely due to the low numbers of litters investigated.

IgM levels in piglets remained below 20 ELISA units (group means) at an age of 2- and 4 weeks of age independent of sow vaccination (Fig. 3B). A highly significant increase of IgM was observed in both groups at the age of 6 weeks compared to previously investigated samples. No significant differences in levels of IgM binding to *S. suis cps*1 were recorded between maternally vaccinated and non-vaccinated piglets.

The course and levels of specific IgA in serum were very similar in both groups (Fig. 3C). At an age of 2 and 4 weeks, litter means remained independent of sow vaccination under 20 and 10 ELISA units, respectively. A significant increase of specific IgA was recorded between 4 and 6-week-old piglets with litter means of 45.5 ELISA units (S.D. = 41.4 ELISA units) for the passively immunized group and 43.6 ELISA units (S.D. = 24.4 ELISA units) for the control group in the sixth week of life.

In this trial, bactericidal assays with reconstituted blood were conducted to read out induction of opsonophagocytosis by maternally-derived antibodies. For serum samples drawn at an age of 2 weeks, the mean survival factor of *S. suis cps*1 was significantly lower for immunized piglets in comparison to non-vaccinated control piglets (76.8 (S.D. = 43.3) and 124.4 (S.D. = 117.4)), respectively. At the age of 6 weeks of life, the mean survival factor of vaccinated-sow piglets was 89.2 (S.D. = 16.7) and not significantly different to the one of control-sow piglets with 121.6 (S.D. = 38.5). Overall, survival factors were very high in these assays indicating proliferation of streptococci.

### Trial 2: Prime-boost vaccination of gilts in quarantine with i.m. versus i.n. second boost vaccination prior farrowing

**Fig. 4:**
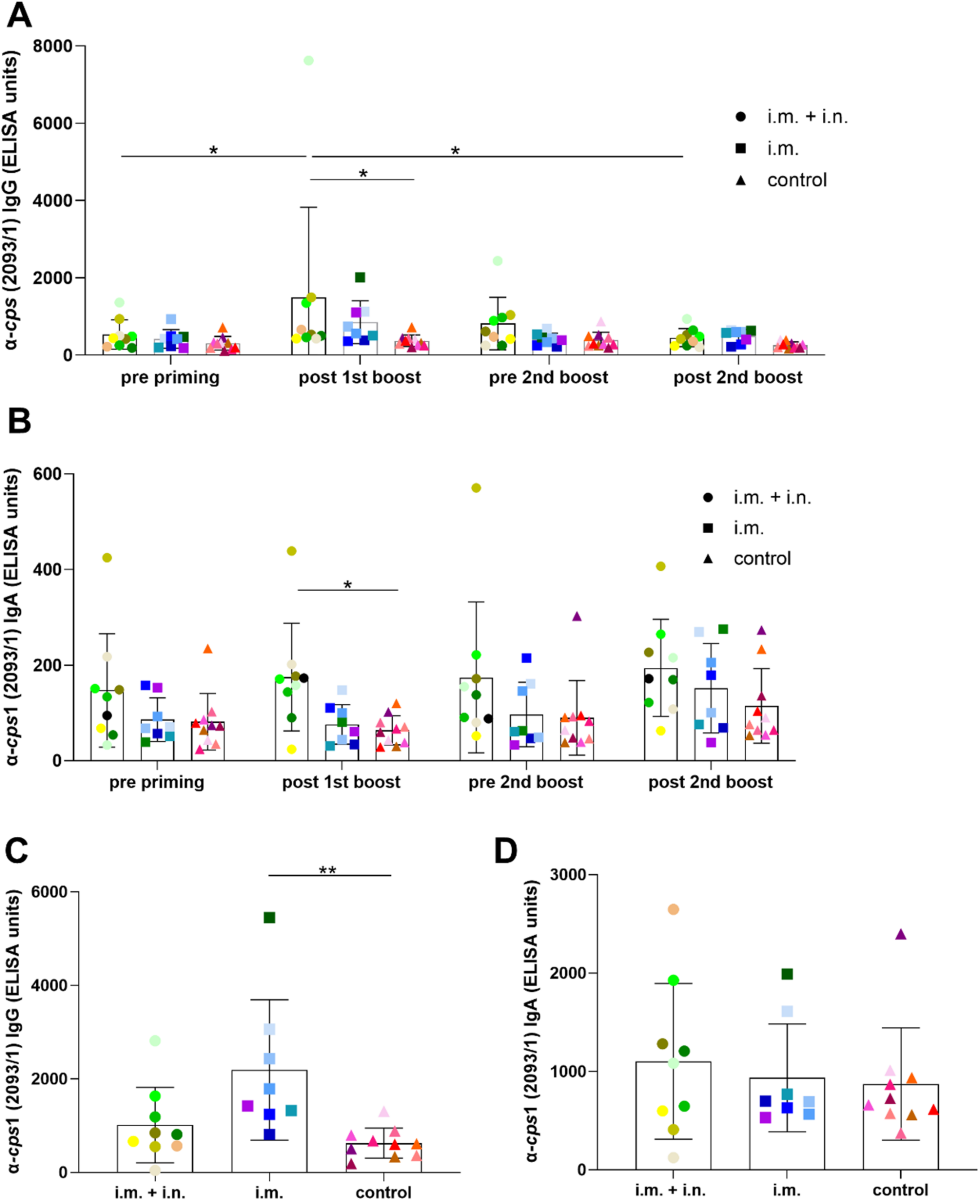
Levels of IgG **(A, C)** and IgA **(B, D)** binding to the surface of *S. suis cps* 1 strain 2093/1 in serum **(A, B)** and colostrum **(C, D)** drawn from gilts of the 2^nd^ trial at the indicated time points (trial 2). Gilts were either immunized intramuscularly 2 times during quarantine and boostered 3 weeks before farrowing (intranasal application: i.m. + i.n., intramuscular application: i.m.) with an autologous *cps*1 vaccine or remained unvaccinated (control). Specific gilts of trial 2 (Fig. 4) and the respective litters (Fig. 5) are indicated by the same color. Serum samples (**A**, **B**) were drawn before priming (pre priming), 2 weeks after the first boost immunization (post 1st boost), 3 weeks (pre 2^nd^ boost) and 1 week (post 2nd boost) before farrowing. Colostrum samples (**C**, **B**) were collected within 12 h after the onset of birth. IgG (**A**,** C**) and IgA (**B**,** D**) levels binding to the surface of *cps*1 strain 2093/1 were determined in a whole cell ELISA. Mean values are indicated by horizontal lines, standard deviations by error bars. Statistical analyses were conducted with the twoway ANOVA and a subsequent Tukey’s multiple comparisons test (**A**, **B**) or Dunn´s multiple comparisons test (**C**, **D**). Significant differences are indicated (* *p* < 0.05, ** *p* < 0.01)

The second trial was conducted with gilts to assess induction of maternally-derived antibodies through prime-boost vaccination during quarantine and a second boost prior to farrowing. Whereas the vaccination during quarantine was conducted intramuscularly in both vaccination groups, one group was boostered intranasally prior to farrowing with the intention to induce not only IgG but also IgA binding to the *S. suis* outbreak strain. Prime-booster vaccination during quarantine was associated with an increase of specific IgG levels in both vaccination groups but differences between the 2 time points were not significant (Fig. 4A). Two weeks post first boost, the level of IgG binding to *cps*1 were significantly higher in the i.m. group than in the control group. Though the mean level of the group later boostered intranasally was also substantially higher, differences to the control gilts were not significant. A substantial decrease of specific IgG was observed between quarantine and the second boost in the i.m. group. Accordingly, differences to the control group in specific serum IgG levels were not observed prior to the second boost. In the other vaccination group, we did not record a comparable decline of IgG levels but rather a larger variation among gilts. After the second boost and one week before farrowing, the mean specific IgG levels of vaccinated gilts reached mean values of 451 ELISA units (S.D. = 233 ELISA units) and 494 ELISA units (S.D. = 170 ELISA units) in the case of intranasal and intramuscular boost vaccination, respectively (Fig. 4A). These values were substantially higher than the specific IgG levels of non-vaccinated gilts (mean of 259 ELISA units, S.D. = 81 ELISA units) but differences between the three groups were not significant (Fig. 4A). At this time point, levels of serum IgA binding to *cps*1 showed means of 194 ELISA units (S.D. = 102 ELISA units), 152 ELISA units (S.D. = 94 ELISA units) and 115 ELISA units (S.D. = 78 ELISA units) in the case of intranasal second boost, intramuscular second boost and non-vaccinated gilts, respectively (Fig. 4B).

Colostrum samples of the gilts of the second trial were collected to directly read out the impact of the different vaccination schedules on the colostral transfer of specific IgG and IgA. Significantly higher levels of specific IgG were found in intramuscularly boostered gilts with mean of 2080 ELISA units (S.D. = 1290 ELISA units) compared to non-vaccinated gilts with mean of 628 ELISA units (S.D. = 316 ELISA units) (Fig. 4C). Intranasally boostered gilts obtained mean specific colostral IgG levels of 1013 ELISA units (S.D. = 807 ELISA units), which were not significantly different to the control gilts. Five of 9 intranasally boostered gilts obtained specific colostral IgA levels above 1000 ELISA units (Fig. 4D). However, specific IgA levels above 1000 ELISA units were observed in only two i.m.-vaccinated gilts and one control gilt. However, differences in colostral IgA-α-*cps*1 levels between the three groups were not significant.

**Fig. 5:**
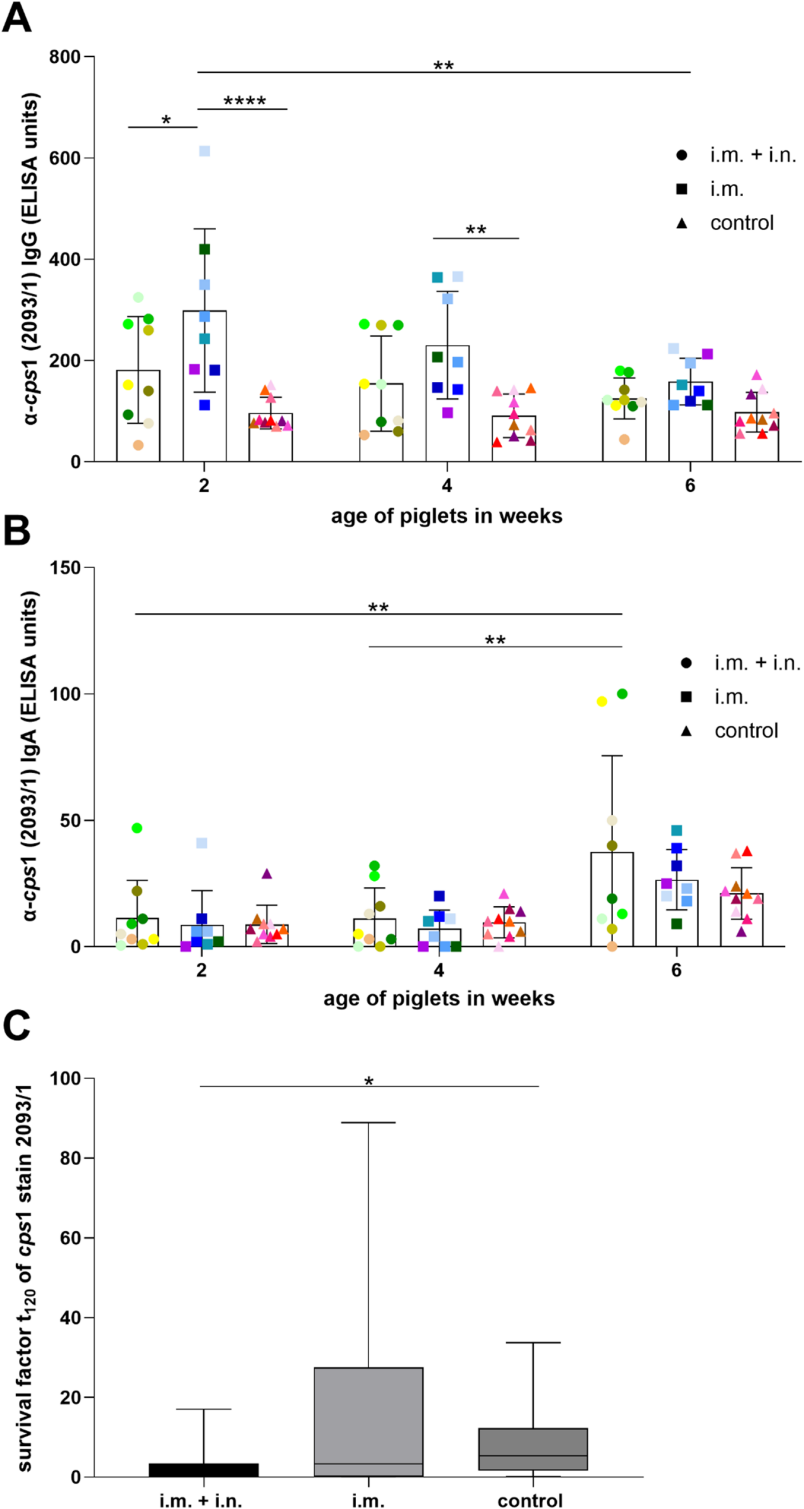
Levels of IgG **(A)** and IgA **(B)** binding to the surface of *S. suis cps*1 in sera drawn from piglets at the indicated age and survival of *S. suis cps*1 **(C)** in blood of these piglets at the age of 2 weeks (trial 2). Piglets were raised by non-vaccinated (control, *n* = 10 gilts) or vaccinated gilts (i.m. + i.n., *n* = 9 gilts; i.m., *n* = 8 gilts) and weaned at an age of 4 weeks. Prime-boost vaccination of gilts was conducted during quarantine prior to fertilization. For each investigated litter from vaccinated and control gilts, two piglets were randomly selected and sampled at 2, 4 and 6 weeks of age. The two sera drawn from two littermates at a specific time point served as duplicate. Litter means were calculated and used as statistical parameter. A specific litter is indicated by the same color in **A** and **B**. IgG (**A**) and IgA (**B**) levels binding to the surface of *cps*1 strain 2093/1 were determined in a whole cell ELISA. Mean values are indicated by horizontal lines, standard deviations by error bars. Statistical analyses were conducted with the two-way ANOVA and a subsequent Tukey’s multiple comparisons test. **C** Two weeks after farrowing heparinized blood samples were drawn from two piglets per litter (8 litters per group for this experiment) to investigate survival of the *S. suis* outbreak strain 2093/1 in blood in vitro (bactericidal assay). The survival factor represents the ratio of CFU of *S. suis cps*1 at 120 min to CFU at time point zero. Survival factors above one indicate proliferation of streptococci. Individual values were used as statistical parameter. Medians and quartiles are indicated by horizontal lines. Statistical analyses were conducted with the Kruskal-Wallis test and a subsequent Dunn´s multiple comparisons test. Significant differences are indicated (* *p* < 0.05, ** *p* < 0.01, **** *p* < 0.0001)

In good correlation with the differences between colostrum samples, significantly higher levels of specific serum IgG were recorded in piglets of intramuscularly boostered gilts at the age of two weeks with mean of 298 ELISA units (S.D. = 161 ELISA units) compared to piglets from intranasally boosterd gilts with mean of 181 ELISA units (S.D. = 105 ELISA units) and piglets from non-vaccinated gilts with mean of 96 ELISA units (S.D. = 31 ELISA units) (Fig. 5A). Significant differences were also observed at 4 weeks of age regarding the i.m. group with mean of 230 ELISA units (S.D. = 106 ELISA units) and the control group with mean of 91 ELISA units (S.D. = 43 ELISA units). Specific serum IgA levels were comparable between the three groups at any of the three investigated time points (Fig. 5B). At an age of 2 weeks only single litters in each group obtained values above 25 ELISA units of specific serum IgA. Of note, an increase of specific IgA levels in serum samples was recorded between 4 and 6 weeks of age.

At an age of 2 weeks, heparinized blood of piglets of the 2^nd^ trial was infected in vitro with the *cps*1 outbreak strain to read out opsonophagocytosis (Fig. 5C). Streptococci proliferated in all samples of the control group (survival factor greater 1), whereas killing of *S. suis cps*1 was documented in 2 of 8 samples in the i.m. group and 4 of 8 samples in the i.m. + i.n. group. However, no significant differences between the groups were recorded with mean survival factors of 2.9 (S.D. = 4.6), 15.4 (S.D. = 19.4) and 9.8 (S.D. = 9) for the i.m. + i.n. group, i.m. group and control group, respectively.

### Trial 3: Evaluation of the second intramuscular booster of gilts pre farrowing

The second trial demonstrated increased specific IgG levels in colostrum of gilts prime-boost vaccinated during quarantine and boostered a second time intramuscularly pre farrowing. This was in agreement with significantly increased specific IgG levels in their litters at the second and fourth week of life. Overall, the results of the second trial suggested that the second boost is important to ensure these high IgG levels. The third trial was designed to specifically address the role of the second boost by comparison to a group of gilts that was only prime boost vaccinated during quarantine. Furthermore, longitudinal milk samples were collected in trial 3 to read out the dynamics of specific immunoglobulins during the suckling period.

**Fig. 6:**
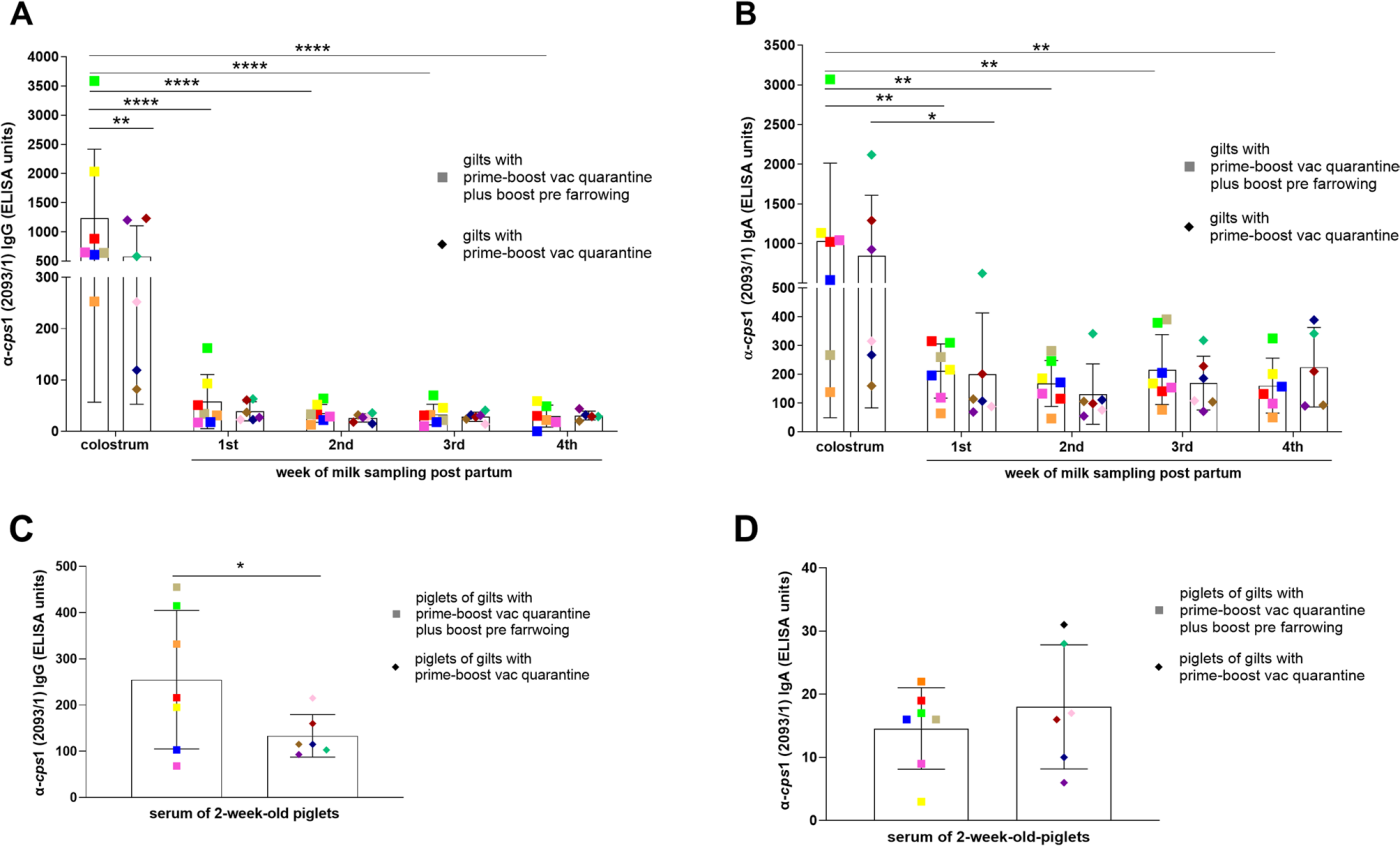
Levels of IgG **(A, C)** and IgA **(B, D)** binding to the surface of *S. suis cps*1 in colostrum and milk samples **(A, B)** collected from prime-boost vaccinated and prime-boost-boost vaccinated gilts and in serum of their 2-week-old-litters **(C, D)** (trial 3). Gilts were prime-boost immunized with a *cps*1 bacterin during quarantine (prior fertilization). Three weeks pre farrowing one group (*n* = 7) obtained a second boost vaccination (gilts with prime boost vac quarantine plus boost pre farrowing) while in the other group (*n* = 6) no second booster was administered (gilts with prime boost vac quarantine). All vaccinations were applied intramuscularly. **A**,** B** Colostrum and milk samples were collected within 12 h after birth and at the indicated time points, respectively. **C**,** D** Two piglets from each litter were randomly chosen, treated as duplicate and sampled at 2 weeks of age. Specific IgG and IgA levels were determined in a whole cell ELISA. A specific gilt and its litter are indicated by the same color. Mean values are indicated by horizontal lines, standard deviations by error bars. Statistical analyses were conducted with the two-way ANOVA and a subsequent Tukeys multiple comparisons test (**A**, **B**), the Mann-Whitney *U*-test (**C**) or the unpaired t-test (**D**). Significant differences are indicated (* *p* < 0.05, ** *p* < 0.01, **** *p* < 0.0001)

Specific IgG levels were approximately twice as high in colostrum samples of gilts boostered pre farrowing with a mean of 1236 ELISA units (S.D. = 1179 ELISA units) in comparison to gilts only prime-boost vaccinated during quarantine with a mean of 578 ELISA units (S.D. = 525 ELISA units) (Fig. 6A). This is in agreement with significantly higher levels of IgG in litters raised by gilts boostered pre farrowing with a mean of 255 ELISA units (S.D. = 150 ELISA units) in comparison to litters of gilts only prime-boost-vaccinated during quarantine with a mean of 133 ELISA units (S.D. = 46 ELISA units) (Fig. 6C).

In agreement with the results of the other trials, specific IgA levels in colostrum were not significantly different between the two groups with means of 1031 ELISA units (S.D. = 982 ELISA units) and 847 ELISA units (S.D. = 763 ELISA units) for the group with and without second boost immunization, respectively. This was also true for the different milk samples and for serum IgA levels of 2-week-old-piglets (Fig. 6B/6D). Levels of specific IgA in milk were approximately one fifth of the level in colostrum and remained relatively stable between the first and fourth week of life. As expected, the difference in IgG levels between colostrum and milk was very high with 10-to-20-fold increased values in colostrum in comparison to milk samples (Fig. 6A).

## Discussion

Worldwide, the most common *cps* of *S. suis* among invasive isolates is *cps*2 [4]. This does, however, not necessarily mean that it is also the most virulent. An older comparative study showed that European *mrp*^s^*epf* + *cps*1 strains are highly virulent and even more virulent than *mrp*^+^*epf* + *cps*2 [19]. In agreement with this publication, we describe here a more recent devastating *cps*1 outbreak of severe diseases among suckling piglets in a farm in Austria. In this farm, mainly suckling piglets were affected. Though differences in age of diseased piglets in association with infection by different *S. suis cps* are overall not well documented, previous studies suggested already that *cps*1 primarily causes disease in suckling and not in weaning or growing piglets, as the case in *cps*2 and *cps*9 [17, 20].

Previous studies demonstrated elevated anti-MRP IgG levels in colostrum and serum from 2-week-old piglets after prime-boost vaccination of sows with an autogenous *S. suis cps*2 bacterin prior to farrowing [11] and increased anti-*S. suis* IgG titers in gilts post boost vaccination with a multi-serotype autogenous bacterin [21] or a *cps*7 autogenous bacterin [22]. The first trial of this study confirms that maternally-derived IgG levels against a *S. suis cps*1 outbreak strain can be increased significantly in 2- and 4-week-old piglets through preparturient *S. suis* bacterin prime-boost vaccination of sows. We used different adjuvants in the prime boost vaccination in trials 1–3 (Emulsigen^®^) in comparison to the second boost vaccination in trial 2 (Montanide™ gel). Montanide™ gel was used as it is recommended for mucosal vaccination by the manufacturer. Of note, various adjuvants have been tested in swine, but further studies are needed to identify adjuvant candidates that lead to a prominent induction of mucosal immunity [23]. Adjuvants have been shown to play an important role in *S. suis* bacterins [7, 9]. There are some contradictory results on the protective efficacy of *S. suis* bacterins including Emulsigen^®^ [7, 8]. However, Emulsigen^®^ was also used as adjuvant in a previous study demonstrating that vaccination of sows with a *cps*2 bacterin pre farrowing is associated with protection against experimental infection against the homologous strain in 6-week-old piglets [11].

The present study was not designed to read out protection against *S. suis* disease. However, data recorded during management of this herd suggests protection: After introduction of the autogenous bacterin the baseline mortality of suckling piglets declined from 12.8 to 11.6%. Furthermore, the antimicrobial use, measured in defined daily dose (DDD) values, amounted to 59,500 in the 6 months prior to the *S. suis* outbreak, 372,250 during the 6 months of the outbreak, and 43,416 in the 6 months after the introduction of bacterin vaccination on this farm (The DDD is an European Medicines Agency standard used across EU countries, including Austria, to quantify veterinary antimicrobial use in pigs, based on the average daily dose (mg) per kg body weight for the main indication). The decreased mortality of suckling piglets, the prominent DDD decline after introduction of bacterin vaccination and the significantly reduced survival factors of *S. suis cps*1 in blood reconstituted with sera from maternally vaccinated versus non-vaccinated, 2-week-old piglets, suggests in agreement with previous studies that maternally-derived antibodies might elicit protection against *S. suis* disease, most likely through induction of opsonophagocytosis [11, 21]. Serum IgG and IgM antibodies play an important role in control of *S. suis* bacteremia [24]. However, as shown here and also in previous studies [21, 22] vaccination of sows or gilts with a *S. suis* bacterin does not result in increased levels of IgM binding to *S. suis*. As levels of IgA and IgM binding to *S. suis cps*1 were not different between litters fostered by vaccinated and non-vaccinated sows (trial 1) or gilts (trial 2, only IgA investigated), we propose that maternally-derived antibodies induced by intramuscular *S. suis* bacterin application are mainly IgG and that protective efficacy elicited by maternity vaccination with *S. suis* bacterins depends mostly on IgG.

Prime-boost-boost vaccination of incoming gilts is a common practice in Europe and North America [21]. Many incoming gilts are placed in quarantine units prior to introduction into the herd and breeding. It is very common to screen and vaccinate these gilts during quarantine. In the second and third trial, we investigated immunogenicities of an autogenous *S. suis cps*1 bacterin applied to gilts during quarantine and boostered a second time pre farrowing. Though this study was conducted in a closed herd, gilts showed IgG levels substantially lower than sows (compare values of trial 1 and 2) and in some cases only a moderate seroconversion was observed. Specifically, 2-week-old piglets of i.m prime-booster-boostered gilts showed mean anti *S. suis cps*1 IgG levels of 298 and 255 ELISA units in trials 2 and 3, respectively. The third trial demonstrated that the second booster in gilts is crucial to ensure mean specific IgG levels above 200 ELISA units in serum of their 2-week-old piglets. Though sows were boostered only once, their 2-week-old piglets had much higher anti *S. suis cps*1 IgG levels (mean of 1003.4 ELISA units). This comparison and the kinetics of IgG levels justify a second booster vaccination in gilts. It might be speculated that two booster vaccinations of gilts pre farrowing are even more effective in induction of high specific IgG levels in their litters.

The vaccination protocol of our trial 2 is similar to the one of the autogenous *S. suis cps*7 bacterin investigated by Corsaut et al. [22]. Though there are similarities in the kinetics of IgG levels in vaccinated gilts to this previous study, a major difference is that we recorded significant differences in 4-week-old piglets, whereas this was only the case in 7-day-old but not 18-day-old piglets in the cited study. This comparison shows that there are many uncertainties associated with autogenous *S. suis* vaccines as differences in adjuvant as well as culture and inactivation of streptococci might have a substantial impact on immunogenicity and protective efficacy [6].

Vaccination of colostrum-deprived piglets with a *S. suis cps*9 bacterin does not influence colonization and transmission [13]. As mucosal IgA plays an important role in immune exclusion of pathogens on the mucosal surface, as shown for example for *Streptococcus pneumoniae* in murine colonization models [25, 26], we envision that a vaccination protocol eliciting elevated levels of specific IgA might have an effect on colonization and transmission of important pathotypes of *S. suis* such as the *cps*1 outbreak strain in this study. We figured that an intranasal booster to gilts already prime-boostered intramuscularly during quarantine might be a way to induce high specific IgG and IgA levels. However, differences in specific IgA levels were not associated with intranasal boost vaccination and levels of specific IgG in colostrum were lower in i.n. compared to i.m. boostered gilts.

Our data overall indicates that vaccination of gilts with the autogenous *S. suis* bacterin exerts very limited if any influence on specific IgA levels in colostrum or milk (Fig. 6B), suggesting that bacterin application may not induce mucosal immunity. This is in agreement with results of Mariela Segura`s group showing that vaccination of gilts with a multi-serotype autogenous *S. suis* bacterin does not have an influence on shedding of any of the investigated serotypes via saliva [21]. Accordingly, we question that vaccination of gilts during quarantine has in comparison to vaccination after breeding a positive influence on the control of *S. suis* disease in an affected herd.

In the first and second trial of this study we observed a prominent increase of IgM and to a lesser extent also of IgA binding to *S. suis cps*1 in piglets between the fourth and sixth week of life. Importantly, levels of these antibodies were not different between maternally-vaccinated and non-vaccinated piglets. This suggests that there is no inhibitory effect of maternally-derived IgG on B cell activation after the fourth week of life leading to secretion of IgM. We have shown through cleavage of IgM with the specific IgM protease Ide_*Ssuis*_ that IgM plays an important role in bactericidal immunity against *S. suis cps*1 in porcine blood in 6-week-old piglets [17]. Thus, it is important to know that sow vaccination does not lead to an inhibition of IgM secretion by B cells post weaning. The increase of IgM after weaning confirms results of previous studies [17, 27], but to the best of our knowledge this is the first study, that presents data indicating that IgA binding to *S. suis* in serum is also increasing between the fourth and sixth week of life. Kinetics of IgM and IgA binding to *S. suis* appear different to kinetics of specific IgG, as piglets of vaccinated and also non-vaccinated sows and gilts did not show an increase of specific IgG in this time period. The latter is in accordance with our previous *cps*1 study [17].

In conclusion, prime-boost vaccination of sows and prime-boost-boost vaccination of gilts with an autogenous *S. suis cps*1 bacterin might lead to significantly increased specific IgG levels in 2- and 4-week-old piglets but does not exert a detectable influence on IgM and IgA levels. Booster vaccination pre farrowing is important to ensure elevated IgG levels in litters of gilts vaccinated during quarantine.

## Conclusions

Intramuscular prime-boost vaccination of sows and gilts with an autogenous *S. suis* serotype 1 bacterin and Emulsigen^®^ is associated with significantly increased levels of specific IgG in colostrum and serum of 2- and 4-week-old piglets based on the investigations in one herd that had experienced a severe *S. suis cps*1 outbreak. A further boost-vaccination of vaccinated gilts pre-farrowing is crucial to ensure increased IgG levels in their piglets. Furthermore, our results suggest that levels of serum IgM and IgA binding to *S. suis cps*1 in sera drawn from 2- to 6-week-old piglets are not influenced by preparturient sow or gilt vaccination with a *S. suis cps*1 bacterin.

## Data Availability

The datasets used and/or analysed during the current study are available from the corresponding author on reasonable request.
